# Association between combined exposure to plasma heavy metals and dyslipidemia in a chinese population

**DOI:** 10.1186/s12944-022-01743-6

**Published:** 2022-12-06

**Authors:** Tingyu Luo, Shiyi Chen, Jiansheng Cai, Qiumei Liu, Ruoyu Gou, Xiaoting Mo, Xu Tang, Kailian He, Song Xiao, Yanfei Wei, Yinxia Lin, Shenxiang Huang, Tingjun Li, Ziqi Chen, Ruiying Li, You Li, Zhiyong Zhang

**Affiliations:** 1 Department of Environmental Health and Occupational Medicine, School of Public Health, Guilin Medical University, No.1 Zhiyuan Road, Guangxi 541199 Guilin, China; 2 Guangxi Health Commission Key Laboratory of Entire Lifecycle Health and Care, Guilin Medical University, Guangxi 541199 Guilin, China; 3 School of Public Health and Management, Guangxi University of Chinese Medicine, Guangxi 530200 Nanning, China; 4 Department of Environmental and Occupational Health, School of Public Health, Guangxi Medical University, 530021 Nanning, Guangxi China

**Keywords:** Dyslipidemia, Heavy metals, Bayesian kernel machine regression

## Abstract

**Background:**

Exposure to heavy metals in the environment is widespread, while the relationship between combined exposure to heavy metals and dyslipidemia is unclear.

**Methods:**

A cross-sectional study was performed, and 3544 participants aged 30 years or older were included in the analyses. Heavy metal concentrations in plasma were based on inductively coupled plasma‒mass spectrometry. The relationship between heavy metals and dyslipidemia was estimated by logistic regression. BKMR was used to evaluate metal mixtures and their potential interactions.

**Results:**

In logistic regression analysis, participants in the fourth quartile of Fe and Zn (Fe > 1352.38 µg/L; Zn > 4401.42 µg/L) had a relatively higher risk of dyslipidemia (Fe, OR = 1.13, 95% CI: 0.92,1.38; Zn, OR = 1.30, 95% CI: 1.03,1.64). After sex stratification, females in the third quartile of plasma Zn (1062.05-4401.42 µg/L) had a higher relative risk of dyslipidemia (OR = 1.75, 95% CI: 1.28, 2.38). In BKMR analysis, metal mixtures were negatively associated with dyslipidemia in females when all metal concentrations were above the 50th percentile. In the total population (estimated from 0.030 to 0.031), As was positively associated with dyslipidemia when other metals were controlled at the 25th, 50th, or 75th percentile, respectively, and As was below the 75th percentile. In females (estimated from − 0.037 to -0.031), Zn was negatively associated with dyslipidemia when it was above the 50th percentile.

**Conclusion:**

This study indicated that As was positively associated with dyslipidemia and that Zn may be negatively associated with dyslipidemia in females. Combined metal exposure was negatively associated with dyslipidemia in females. Females with low plasma Zn levels are more likely to develop dyslipidemia and should receive more clinical attention in this population.

## Background

Abnormalities in the metabolism of lipid levels in human blood are known as dyslipidemia. It is one of the common risk factors and predictors of cardiovascular disease [1], and triglycerides (TGs), total cholesterol (TC), low-density lipoprotein cholesterol (LDL-C) and high-density lipoprotein cholesterol (HDL-C) are indicators of dyslipidemia [2]. In 2018, the prevalence of dyslipidemia in the United States was approximately 56.8%, while in China, it was 42.7%. Insufficient attention to the rapidly growing phenomenon of dyslipidemia and poor treatment and control effects lead to the increased global burden [3]. Something must be made to prevent and control dyslipidemia, as it is a significant global public health problem.

Dyslipidemia is caused by a combination of environmental and genetic factors [4]. Widely distributed heavy metals in the environment are considered to be risk factors for dyslipidemia [5]. People can easily be exposed to heavy metals in the environment through water, air, and soil. Heavy metals are difficult to degrade and tend to accumulate [6]. Therefore, heavy metal pollution has become a very important environmental problem at present. The heavy metals chromium (Cr), iron (Fe), zinc (Zn), arsenic (As), strontium (Sr), cadmium (Cd), and lead (Pb) may be associated with dyslipidemia occurrence [3, 7]. People are often exposed to heavy metals in their daily lives through exposure to industrial waste gases, wastewater, contaminated soil, drinking water, or food [8]. Pollution is caused by metal mines, smelting, and others. Different living habits are also sources of heavy metal pollution, such as smoking and eating raw meat [9–12]. Chromium (III), Fe and Zn are essential trace elements in humans and they promote lipid metabolism in vivo [13, 14]. Supplementation with these elements reduced HDL-C concentrations and improved dyslipidemia in rats [15]. In addition, Sr and Cd can interfere with the antioxidant activity of normal cells and promote hyperlipidemia in mice [16, 17]. Exposure to As and Pb stimulates lipid peroxidation in vivo, increasing triglyceride levels and even leading to hyperlipidemia in mice [18, 19]. Different levels of metal exposure may affect the accuracy of the results of association analysis between heavy metals and dyslipidemia. Most of the available reports explore the effects of single metals on dyslipidemia. However, the combined effects of combined multimetal exposures on humans are closer to the true state.

Bayesian kernel machine regression (BKMR) is a machine learning method for assessing the health effects of multiple concurrent exposures. This method explains the composition of the mixture, systematically treats highly correlated exposures, and identifies important components in the mixture [20]. The method uses an estimated exposure response function to more realistically estimate the exposure-response relationship among mixtures, thereby capturing potential interactions among combined exposures [21]. Currently, many researchers use the BKMR to assess the health effects of multiple environmental confounders [22–24].

The BKMR model in this study was used to investigate the relationship between the levels and combined effects of Cr, Fe, Zn, As, Sr, Cd, and Pb in the plasma of ordinary people and dyslipidemia to provide a reference for the prevention and treatment of dyslipidemia.

## Materials and methods

### Study participants

A cross-sectional study with physical examinations and questionnaires among rural adults aged 30 years and older from 2018 to 2019 in Gongcheng Yao Autonomous County, Guangxi, China. A total of 4356 adults participated in this study. Exclusion criteria for participants in this study were as follows: (a) failed to complete the questionnaire or lacked data on the covariates in the questionnaire; (b) lacked data on height, weight, blood pressure at rest (systolic/diastolic), plasma TG, TC, HDL-C, LDL-C and glucose (GLU); (c) took antihyperlipidemic drugs; and (d) soutliers greater than 3 times the 99th percentile of the metal detection limit. Eventually, 3544 participants were left for analysis. The Ethics Committee of Guilin Medical University approved the project (No. 20180702-3). Each participant signed written informed consent.

### Measurement of plasma metal concentration

Before the morning physical examinations, participants were required to fast for 12 h. The collected blood samples were centrifuged at 4 °C, and the upper plasma layer was taken. Plasma samples were cryopreserved at -80 °C before testing. First, a 1% nitric acid dilution was configured using 65% concentrated HNO_3_ (65% HNO_3_, superior pure), 0.01% Triton X-100 (Merck Inc., Dam Satart, Germany) and 0.5% n-butanol (Thermofisher, Reagent Lane, Fair Lawn, NJ 07410, USA) at a ratio of 100:1:50 diluted with ultrapure water. Then 0.1 mL of plasma was taken and diluted to 2.0 mL with 1% nitric acid diluent. The content of metals in standard controls (ClinChek® Level1 and Level2; Recipe Chemicals; Germany) was used for quality control, and it was verified while the tested metal concentrations were within the reference concentration value of the reagent instruction manual. Inductively coupled plasma‒mass spectrometry (Thermo Fisher Scientific iCAPRQ) was used to measure the metal content in plasma. The intraanalytical and interanalytical coefficients for changes in plasma metal content were all less than 10%. The spiked method (standard addition) was used along with the internal standard method to determine the spiked recoveries. The spiked recoveries of all samples were between 80.2 and 115. The limits of detection (LODs) of Cr, Fe, Zn, As, Sr, Cd, and Pb were 0.019, 0.021, 0.071, 0.005, 0.018, 0.002, and 0.015 µg/L, respectively. All plasma metals were measured above the LOD.

### Dyslipidemia detection and definition

Blood samples were collected and sent to the local hospital’s laboratory department the same morning to test TC, TG, HDL-C and LDL-C lipid profiles according to uniform standards. The indicators of dyslipidemia in this study refer to the relevant criteria in the Guidelines for the Management of Dyslipidemia in Chinese Adults [2]: TC ≥ 6.22 mmol/L; TG ≥ 2.26 mmol/L; LDL-C ≥ 4.14 mmol/L; and HDL-C < 1.04 mmol/L. Meeting one or more of the above conditions and/or taking lipid-lowering medications was considered to be a dyslipidemia occurrence.

### Definition of covariates

Investigators were uniformly trained to collect information on sex (male or female), age (< 60 or ≥ 60 years), ethnicity (Han, Yao, or other), education (≤ 6 or > 6 years), smoking (yes or no), alcohol consumption (yes or no), fasting blood glucose, blood pressure at rest (systolic/diastolic), and body mass index (BMI) from each participant’s questionnaire. Participants who smoked at least one cigarette per day were defined as smokers. Drinkers were defined as those who drank more than 50 g of alcohol at least once a month. Participants whose fasting blood glucose concentrations were ≥ 7.0 mmol/L or who were taking hypoglycemic medications were considered hyperglycemic. Participants whose blood pressure ≥ 140/90 mmHg or who were taking blood pressure-lowering medication were considered hypertensive [25]. BMI [weight (kg)/square of height (m^2^)] was classified as normal weight, overweight (23.0 kg/m^2^ ≤ BMI < 27.5 kg/m^2^) and obese (BMI ≥ 27.5 kg/m^2^) [26].

### Statistical analyses

Frequencies (proportions) in the analysis of this study were used to indicate categorical variables. The mean (SD) ± standard deviation or median quartile (IQR) denoted continuous variables in the analysis of this study. The chi-square test or Wilcoxon rank sum test was used to determine differences between the influencing factors and dyslipidemia. Metal element concentrations were log_10_ transformed before the BKMR analysis to reduce skewness. Logistic regression analysis was used. Sex, age, ethnicity, education, smoking, alcohol consumption, hypertension, hyperglycemia, and BMI were adjusted as covariates when participants were divided into four groups (Q1 to Q4) by metal concentration, with the Q1 group regarded as the reference. The association between exposure levels to the seven metals and dyslipidemia was estimated based on adjusted odds ratios (ORs) with their 95% confidence intervals.

BKMR analyzed the overall association between coexposure to chromium, iron, zinc, arsenic, strontium, cadmium, and lead and dyslipidemia by controlling for the effects of potential nonlinear relationships, and assessed potential interactions between heavy metals and dyslipidemia. The Markov chain Monte Carlo algorithm was applied to calculate the metal-specific exposure-response curves for 50,000 iterations. The metal-to-metal interaction effects in the model were then validated by using traditional logistic regression interaction analysis methods. The BKMR-adjusted covariates were consistent with the binary logistic regression analysis.

Sex stratification was applied to logistic regression and BKMR models to analyze the effect of combined metal exposure on dyslipidemia under different metal exposure conditions. All data were obtained using IBM SPSS statistical software, version 24.0 (SPSS Inc., Chicago, Illinois), and R software, version 4.1.2. A *p*- value of < 0.05 on both sides was considered statistically significant.

## Results

### Characteristics of participants

The demographic characteristics of 3544 study participants are shown in Table 1. Among them, there were 1330 males (37.5%) and 2214 females (62.5%), with an average age of 57.51 ± 12.10 years. The Yao population was the largest, accounting for 74.9% of the total; 64.2% had less than 6 years of education. The average blood glucose and BMI of the study participants were 4.96 ± 1.29 mmol/l and 22.7 ± 3.30 kg/m^2^, respectively. Participants with dyslipidemia tended to be older and had higher blood pressure, glucose levels and BMI than those without dyslipidemia. Sex, education, smoking and alcohol consumption were not associated with the occurrence of dyslipidemia.

**Table 1:** Descriptive characteristics of participants

	Total(*n* = 3544)	Nondyslipidemia	Dyslipidemia	*P*
**Sex**				0.25^a^
Male	1330 (37.5)	819 (36.8)	511 (38.7)	
Female	2214 (62.5)	1406 (63.2)	808 (61.3)	
**Age**	57.51 ± 12.10	56.70 ± 12.69	58.88 ± 10.89	< 0.01^a^
< 60years	1770 (49.9)	1168 (52.5)	602 (45.6)	< 0.01^b^
≥ 60years	1774 (50.1)	1057 (47.5)	717 (54.4)	
**Ethnicity**				< 0.01^b^
Han	715 (20.2)	403 (18.1)	312 (23.7)	
Yao	2654 (74.9)	1701 (76.4)	953 (72.3)	
other	175 (4.9)	121 (5.4)	54 (4.1)	
**Education**				0.74^b^
≤ 6years	2274 (64.2)	1423 (64.0)	851 (64.5)	
> 6years	1270 (35.8)	802 (36.0)	468 (35.5)	
**Smoking status**				0.50^b^
Yes	649 (18.3)	400 (18.0)	249 (18.9)	
No	2895 (81.7)	1825 (82.0)	1070 (81.1)	
**Drinking status**				0.46^b^
Yes	1129 (31,9)	699 (31.4)	430 (32.6)	
No	2415 (68.1)	1526 (68.6)	889 (67.4)	
**Hypertension**				< 0.01^b^
Yes	1622 (45.8)	897 (40.3)	725 (55.0)	
No	1922 (54.2)	1328 (59.7)	594 (45.0)	
**Hyperglycemia**	4.96 ± 1.29	4.85 ± 1.21	5.14 ± 1.41	< 0.01^b^
Yes	177 (5.0)	82 (3.7)	95 (7.2)	
No	3367 (95.0)	2143 (96.3)	1224 (92.8)	
**BMI kg/m**^**2**^	22.7 ± 3.30	22.16 ± 3.09	23.62 ± 3.44	< 0.01^a^
< 23	2065 (58.3)	1463 (65.8)	602 (45.6)	< 0.01^b^
23-27.5	1195 (37.7)	641 (28.8)	554 (42.0)	
≥ 27.5	284 (8.0)	121 (5.4)	163 (12.4)	

Data are presented as n (%), mean ± SD or median quartile (IQR)

^a^Calculated using the chi-square test; ^b^Calculated using the rank sum test

The distribution characteristics of the seven metal concentrations in different populations are shown in Table 2. The concentrations of Cr, Fe, Zn, As, Sr and Pb in plasma differed between the control and diseased groups in all participants (*P* < 0.05). Plasma concentrations of Fe in males differed between the control and diseased groups (*P* < 0.05). The plasma concentrations of Cr, Zn, As, Sr and Pb in females differed between the control and diseased groups (*P* < 0.05).

**Table 2:** Distribution characteristics of plasma heavy metals in different populations

	Nondyslipidemia	Dyslipidemia	*P*
**Total**			
Cr (µg/L)	3.05 (1.98,4.15)	2.88 (1.81,3.99)	< 0.01
Fe (µg/L)	1058.81 (808.18,1338.57)	1109.97 (838.60,1362.91)	< 0.05
Zn (µg/L)	1114.34 (764.19,5081.41)	1017.17 (765.32,2757.71)	< 0.01
As (µg/L)	1.18 (0.91,1.79)	1.24 (0.94,2.26)	< 0.01
Sr (µg/L)	27.22 (21.71,33.65)	26.04 (20.76,32.68)	< 0.01
Cd (µg/L)	0.19 (0.13,0.28)	0.18 (0.13,0.28)	0.07
Pb (µg/L)	5.30 (3.36,8.70)	4.91 (3.23,8.57)	< 0.05
**Male**			
Cr (µg/L)	2.98 (1.88,4.09)	2.89 (1.80,3.98)	0.23
Fe (µg/L)	1177.24 (923.17,1488.81)	1227.94 (989.16,1523.50)	< 0.05
Zn (µg/L)	1071.33 (757.22,4988.00)	1059.65 (775.99,3702.13)	0.58
As (µg/L)	1.23 (0.94,1.74)	1.26 (0.96,2.10)	0.18
Sr (µg/L)	28.13 (22.11,34.62)	27.34 (21.02,34.91)	0.24
Cd (µg/L)	0.19 (0.13,0.29)	0.18 (0.12,0.30)	0.25
Pb (µg/L)	5.79 (3.64,9.19)	5.54 (3.54,9.34)	0.65
**Female**			
Cr (µg/L)	3.09 (2.07,4.19)	2.91 (1.82,3.99)	< 0.01
Fe (µg/L)	1003.81 (758.99,1251.67)	1006.65 (766.15,1248.56)	0.38
Zn (µg/L)	1127.92 (766.02,5138.50)	978.58 (762.01,2175.95)	< 0.01
As (µg/L)	1.14 (0.89,1.86)	1.23 (0.93,2.37)	< 0.01
Sr (µg/L)	26.77 (21.40,33.06)	25.35 (20.60,31.55)	< 0.01
Cd (µg/L)	0.19 (0.14,0.27)	0.18 (0.13,0.26)	0.13
Pb (µg/L)	5.07 (3.20,8.41)	4.56 (3.06,7.99)	< 0.05

Data are presented as the median (IQR)

### Binary logistic regression analysis

As shown in Table 3, iron and zinc levels were associated with dyslipidemia after adjusting for sex, age, ethnicity, education, smoking, alcohol consumption, hypertension, hyperglycemia, and BMI. The ORs for Fe and Zn were 1.12 (95 CI%=0.92–1.38, *P* < 0.05) and 1.30 (95 CI%: 1.03–1.64, *P* < 0.05), respectively, in the Q4 group. After sex stratification, the ORs in the Q4 group of Zn were 1.40 times higher (95 CI%: 1.04–1.89, *P* < 0.05) than those in the Q1 group. This suggests that in females, the occurrence of dyslipidemia is associated with the concentration of Zn.

**Table 3:** Binary logistic regression analysis of plasma heavy metals

		**OR(95%CI)**		
**Variable**	**n**	**Total**	**Male**	**Female**
**As (µg/L)**				
Q1 (≤ 0.92)		1 [Reference]	1 [Reference]	1 [Reference]
Q2 (0.92–1.20)	888	1.13 (0.89,1.42)	1.22 (0.81,1.82)	1.11 (0.82,1.49)
Q3 (1.20–1.97)	886	1.13 (0.92,1.39)	1.20 (0.85,1.71)	1.12 (0.86,1.46)
Q4 ( > 1.97)	888	1.11 (0.91,1.37)	1.10 (0.78,1.55)	1.10 (0.85,1.43)
* P* value for trend	882	0.64	0.72	0.85
**Fe (µg/L)**				
Q1 (≤ 821.05)	886	1 [Reference]	1 [Reference]	1 [Reference]
Q2 (821.05-1074.72)	886	0.86 (0.69,1.06)	0.81 (0.56,1.18)	0.89 (0.68,1.18)
Q3 (1074.72-1352.38)	886	0.88 (0.72,1.08)	0.86 (0.62,1.19)	0.90 (0.68,1.19)
Q4 ( > 1352.38)	886	1.13 (0.92,1.38)	1.16 (0.86,1.56)	1.14 (0.86,1.51)
* P* value for trend		0.03	0.23	0.20
**Zn (µg/L)**	886			
Q1 (≤ 764.59)	886	1 [Reference]	1 [Reference]	1 [Reference]
Q2 (764.59-1062.05)	886	1.19 (0.92,1.53)	1.05 (0.68,1.63)	1.50 (1.08,2.08)
Q3 (1062.05-4401.42)	886	1.47 (1.16,1.87)	1.28 (0.86,1.89)	1.75 (1.28,2.38)
Q4 ( > 4401.42)		1.30 (1.03,1.63)	1.36 (0.93,2.00)	1.40 (1.04,1.89)
* P* value for trend	886	0.01	0.28	0.01
**Cr (µg/L)**	886			
Q1 (≤ 1.90)	886	1 [Reference]	1 [Reference]	1 [Reference]
Q2 (1.90-3.00)	886	0.79 (0.64,0.98)	0.75 (0.52,1.08)	0.83 (0.64,1.08)
Q3 (3.00-4.08)		0.90 (0.73,1.11)	0.99 (0.70,1.40)	0.83 (0.63,1.08)
Q4 ( > 4.08)	886	0.85 (0.69,1.06)	0.71 (0.50,1.00)	0.97 (0.73,1.28)
* P* value for trend	886	0.18	0.09	0.35
**Sr (µg/L)**	887			
Q1 (≤ 21.30)	885	1 [Reference]	1 [Reference]	1 [Reference]
Q2 (21.30-26.82)		1.12 (8.91,0.40)	1.29 (0.89,1.86)	1.02 (0.76,1.36)
Q3 (26.82–33.35)	886	0.93 (0.75,1.15)	0.82 (0.58,1.17)	1.00 (0.76,1.33)
Q4 ( > 33.35)	886	0.91 (0.74,1.12)	0.85 (0.61,1.18)	0.96 (0.73,1.27)
* P* value for trend	886	0.17	0.05	0.98
**Cd (µg/L)**	886			
Q1 (≤ 0.13)		1 [Reference]	1 [Reference]	1 [Reference]
Q2 (0.13–0.19)	886	0.96 (0.76,1.21)	0.98 (0.67,1.45)	0.93 (0.69,1.27)
Q3 (0.19–0.28)	886	1.00 (0.81,1.23)	0.96 (0.68,1.36)	1.03 (0.78,1.35)
Q4 ( > 0.28)	886	0.82 (0.66,1.00)	0.73 (0.51,1.03)	0.90 (0.69,1.18)
* P* value for trend	886	0.18	0.27	0.73
**Pb (µg/L)**				
Q1 (≤ 3.31)	886	1 [Reference]	1 [Reference]	1 [Reference]
Q2 (3.31–5.18)	886	0.95 (0.75,1.21)	0.85 (0.57,1.29)	0.99 (0.73,1.34)
Q3 (5.18–8.64)	886	0.99 (0.80,1.23)	0.82 (0.58,1.17)	1.06 (0.80,1.40)
Q4 ( > 8.64)	886	0.92 (0.75,1.13)	0.82 (0.59,1.14)	1.00 (0.76,1.32)
* P* value for trend		0.85	0.61	0.95

Take Q1 as a reference

^a^Adjusted for sex, age, ethnicity, education, smoking, drinking, BMI, hypertension, hyperglycemia

^b^Stratified by sex, adjusted for age, ethnicity, education, smoking, drinking, BMI, hypertension, hyperglycemia

### BKMR analysis

The posterior probability of inclusion (PIP) values for each metal exposure are shown in Table 4. Zn contributed the most to the total population (PIP = 0.8933) and to females (PIP = 0.9743).

**Table 4:** PIP for conditional inclusion into dyslipidemia by using the BKMR model

**Metal (µg/L)**	**PIP**		
	**Total**	**Men**	**Woman**
**Cr**	0.0321	0.1145	0.0172
**Fe**	0.1542	0.1833	0.0262
**Zn**	0.8933	0.2678	0.9743
**As**	0.4504	0.2275	0.1442
**Sr**	0.1138	0.2877	0.0246
**Cd**	0.0159	0.1068	0.0295
**Pb**	0.0271	0.0565	0.0571

Bayesian nuclear machine regression estimates were used while adjusting for sex, age, ethnicity, education, smoking, alcohol consumption, hypertension, hyperglycemia and BMI

Bayesian nuclear machine regression estimates were used while adjusting for sex, age, ethnicity, education, smoking, alcohol consumption, hypertension, hyperglycemia and BMI.

**Fig. 1:**
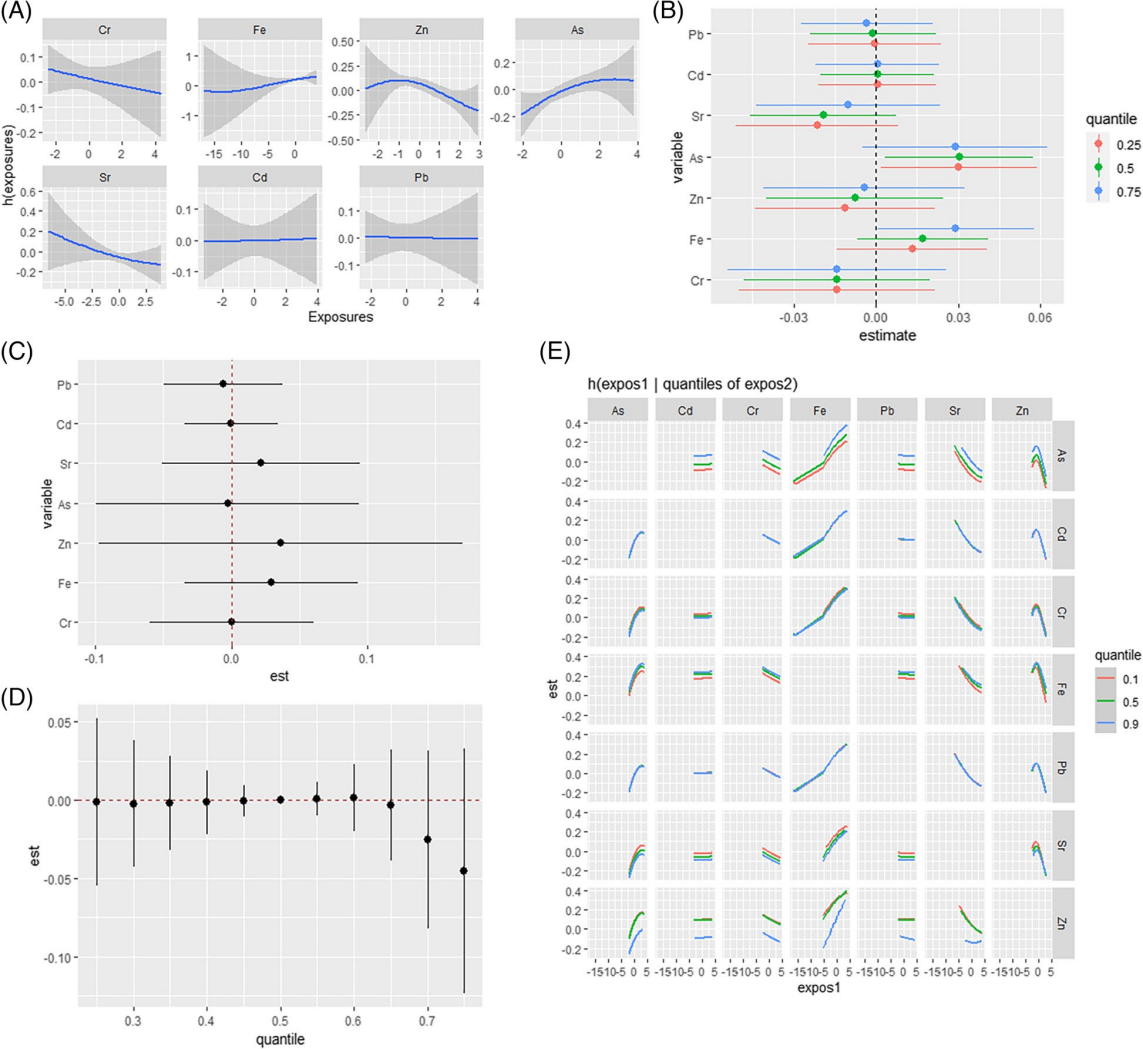
BKMR results in all participants. **A** Univariate exposure-response. **B** Single-exposure effect. **C** Interactive effect. **D** Over effect. **E** Bivariate exposure–response. Data were estimated by Bayesian kernel machine regression while adjusting for sex (male/female), age (30–59 years/≥60 years), race (Han/Yao/other), education (≤ 6 years/>6 years), smoking (yes/no), alcohol consumption (yes/no), BMI (< 23/23-27.49/≥27.5 kg/m^2^), hypertension (yes/no), and hyperlipidemia (yes/no)

In all participants, nonlinear exposure-response relationships were explored by controlling the metal concentrations to maintain corresponding median concentrations. As shown in Fig. 1(A), all exposure response relationships were approximately linear. After fixing all other metals at a specific percentile (25th, 50th, or 75th), As was shown to be positively associated with dyslipidemia when the concentrations were below the 75th percentile (confidence interval not containing 1 at 25th estimate = 0.030; at 50th estimate = 0.031), as shown in Fig. 1(B). As shown in Fig. 1(C), compared with the health risk of a single exposure when all other metals were controlled at the 75th and 25th percentiles, no metal-metal interactions were found. An overall association analysis of the metal mixtures was also performed by controlling metal concentrations at the 50th percentile. It is shown in Fig. 1(D) that the exposure of the metal mixture did not affect the overall subject dyslipidemia. Figure 1(E) shows the binary exposure response function results. A possible interaction was found between both Zn and Sr and Fe. The slope of Fe increased with increasing Zn and Sr levels from the 25th to the 75th percentile.

**Fig. 2:**
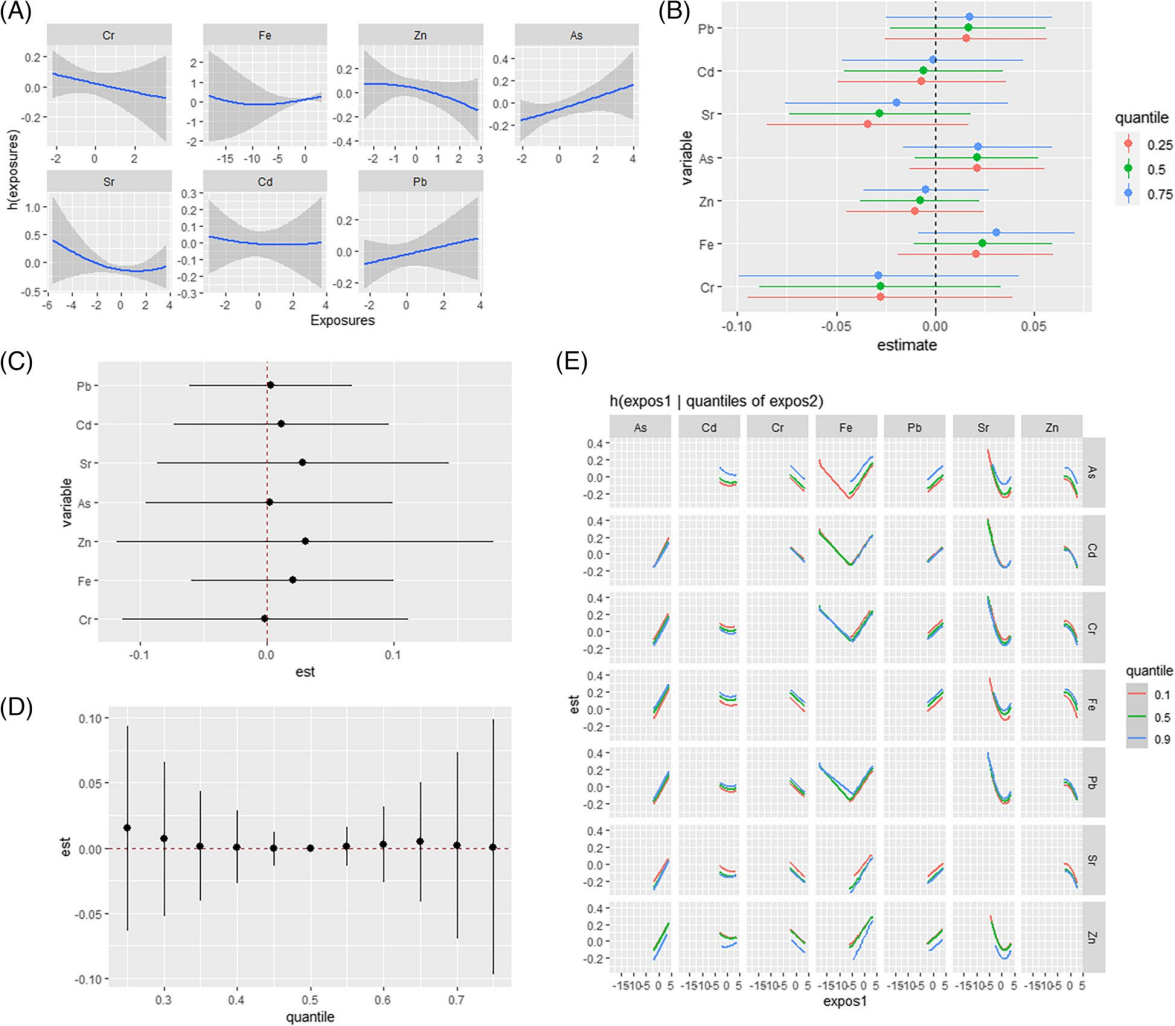
BKMR results in males. **A** Univariate exposure-response. **B** Single-exposure effect. **C** Interactive effect. **D** Over effect. **E** Bivariate exposure–response. Data were estimated by Bayesian kernel machine regression while adjusting for sex (male/female), age (30–59 years/≥60 years), race (Han/Yao/other), education (≤ 6 years/>6 years), smoking (yes/no), alcohol consumption (yes/no), BMI (< 23/23-27.49/≥27.5 kg/m^2^), hypertension (yes/no), and hyperlipidemia (yes/no)

In males, nonlinear exposure-response relationships were explored by controlling the metal concentrations at maintained median concentrations, as shown in Fig. 2(A). All exposure response relationships were approximately linear. After fixing all other metals at a specific percentile (25th, 50th, or 75th), the exposure to metals did not affect dyslipidemia, as shown in Fig. 2(B). As shown in Fig. 2(C), compared with the health risk of a single exposure when all other metal concentrations were controlled at the 75th and 25th percentiles, respectively, no metal-metal interactions were found. An association analysis was performed for all males by fixing the concentration of all metals at the 50th percentile for metal mixtures. As shown in Fig. 2(D), exposure to the mixture of metals had no effect on dyslipidemia in males. A binary exposure response function is shown in Fig. 2(E). Possible interactions were found between Zn and Fe and between Sr and Fe. As the Zn and Sr levels increase from the 25th percentile to the 75th percentile, the slope of Fe also increases.

**Fig. 3:**
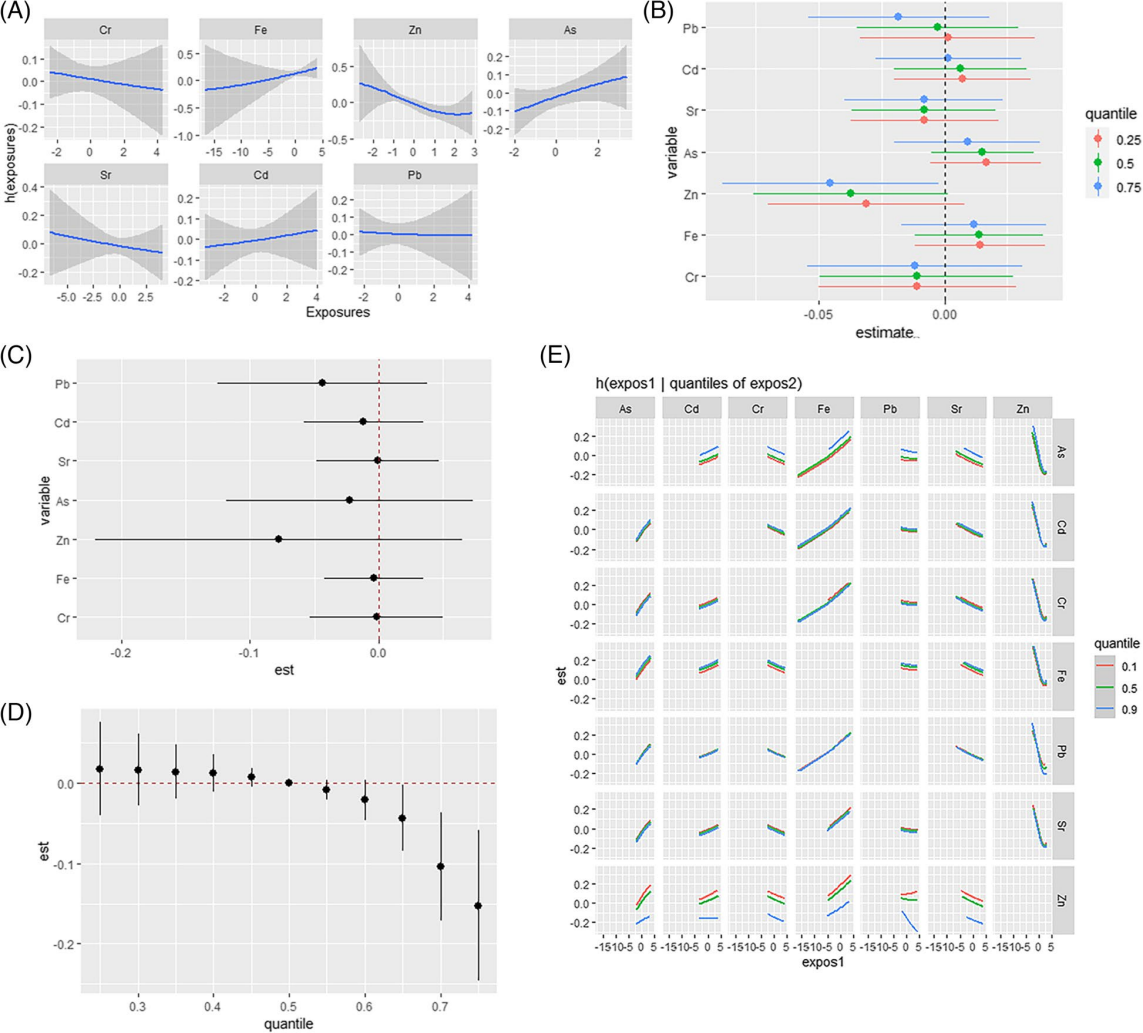
BKMR results in females. **A** Univariate exposure-response. **B** Single-exposure effect. **C** Interactive effect. **D** Over effect. **E** Bivariate exposure–response. Data were estimated by Bayesian kernel machine regression while adjusting for sex (male/female), age (30–59 years/≥60 years), race (Han/Yao/other), education (≤ 6 years/>6 years), smoking (yes/no), alcohol consumption (yes/no), BMI (< 23/23-27.49/≥27.5 kg/m^2^), hypertension (yes/no), and hyperlipidemia (yes/no)

In females, nonlinear exposure-response relationships were explored by controlling the metal concentrations to maintain concentrations. As shown in Fig. 3(A), all exposure response relationships were approximately linear. After fixing all other metals at a specific percentile (25th, 50th, or 75th), Zn was negatively associated with dyslipidemia, and Zn (confidence interval not containing 1 for 50th at estimate = -0.037; 75th at estimate from − 0.031) was higher than the 50th percentile, as shown in Fig. 3(B). As shown in Fig. 3(C), compared with the health risk of a single exposure when all other metal concentrations were controlled at the 75th and 25th percentiles, no metal-to-metal interactions were found. An association analysis was performed for all females by fixing the concentration of all metals at the 50th percentile for metal mixtures. As shown in Fig. 3(D), a negative association was found between the mixture of metals and dyslipidemia when all metal concentrations were above the 50th percentile. Figure 3(E) shows the binary exposure response function results. Possible interactions were found between Zn and Fe and between Zn and As. The slopes of Fe and As increase as the level of Zn increases from the 25th to the 75th percentile.

## Discussion

This study focused on assessing the relationship between plasma mixtures of Cr, Fe, Zn, As, Sr, Cd, and Pb metals and dyslipidemia and tapping into potential metal interactions in participants. The relationship between the seven metals and dyslipidemia was first assessed by binary logistic regression analysis. A significant association was found between Fe and Zn quartile concentrations and dyslipidemia, and a significant association was found between the quartile concentration of Zn and dyslipidemia in females. Then, the combined exposure effects were estimated using the BKMR model, addressing the limitations of the complex interactions that may exist between metals and the possible nonlinear and nonadditive relationships between metals and dyslipidemia [27]. The BKMR model results showed that in the total population, As in the metal mixture was positively associated with dyslipidemia. In females, Zn in the metal mixture was inversely associated with dyslipidemia. Seven metal mixtures had a negative combined effect on female dyslipidemia when all metal concentrations were above the 50th percentile; Zn (PIP = 0.9743) had the largest contribution.

Chronic and acute As exposure induced other diseases, such as cardiovascular, neurological, and metabolic diseases [28]. The prevalence of dyslipidemia increased with increasing levels of As exposure. As exposure was observed in a Taiwanese birth cohort to promote atherogenic lipid metabolism in adolescents [29]. In another cohort study, the prevalence of dyslipidemia was associated with elevated As concentrations in plasma [30]. This result agreed with the present study. However, in the studies by Osorioyanez et al. and Mendez et al., low arsenic exposure did not cause disorders of lipid metabolism and was not significantly associated with dyslipidemia [31, 32]. This may be due to differences in the populations studied and the environment in which the populations lived. Chronic low-dose As exposure can downregulate adiponectin mRNA expression, increase fasting fat production, and promote plasma TG levels, thereby eventually leading to the development of dyslipidemia [33, 34]. Exposure can also reduce plasma HDL levels and increase LDL levels by affecting lipid metabolism [35]. Plasma assays in mice consistently exposed to low As revealed decreased HDL-C/LDL-C ratio levels and increased TG and TC levels [36]. Therefore, As exposure may be a risk factor for dyslipidemia.

Zn may be related to dyslipidemia occurrence. An association between plasma zinc levels and lipid metabolism was found in a meta-analysis [37]. Another meta-analysis that included nine case‒control studies found that Zn reduced TG and TC levels and increased HDL-C levels. Clinically, zinc supplementation has an auxiliary effect on the treatment of dyslipidemia, which can elevate the level of HDL-C and reduce the levels of TC, TG and LDL-C [38]. Zinc-related adipokines can increase lipolysis and reduce adipogenesis in mouse adipose tissue by inhibiting fatty acid synthase (FAS) and diacylglycerol transferase 1 (DGAT1) activity and increasing the activity of hormone-sensitive lipase (HSL) [39]. Zn is a binding site in the zinc-α2-glycoprotein (ZAG) structure and affects lipid metabolism by influencing the binding of ZAG to hydrophobic ligands, especially polyunsaturated fatty acids [40]. Zn reduces the production and release of free fatty acids in adipose tissue by activating signal transduction; it decreases LDL and TG production [41]. Zn supplementation improved lipid levels, reduced plasma TG and LDL, and increased HDL concentrations in type 2 diabetic rats [42]. Clinical Zn supplementation in overweight type 2 diabetic patients [43] showed an improvement in lipid-related indicators. According to the results of the BKMR model in this study, Zn was negatively associated with dyslipidemia levels in females to some extent. Peace N. Ani et al. also found a possible sex-specific association between Zn and dyslipidemia. Gender affects the expression of Zn transporters in vivo [44]. In female patients with dyslipidemia, plasma Zn was negatively associated with dyslipidemia [45]. However, this study did not demonstrate a significant association between sex-heavy metal interactions and dyslipidemia. Hence, the effect of sex on the relationship between Zn and dyslipidemia needs to be further investigated.

Blood levels of iron and chromium have been linked to dyslipidemia in people who are exposed to heavy metals in daily life and work [7, 46, 47]. Combined exposure to Cd, Sr, and Pb is a risk factor for dyslipidemia [48]. However, in this study, a significant association was not found between the above five metal elements and dyslipidemia. Studies on the relationship between heavy metal concentrations in human blood and dyslipidemia are scarce. In a cross-sectional case‒control study, the risk of dyslipidemia increased with elevated blood levels of iron (> 110 µg/L in males; >200 µg/L in females) [49]. Total cholesterol levels were significantly associated with elevated plasma Cr (3.24 µg/L) levels in residents near petrochemical plants [7]. In a case‒control study of Sr and T2D risk, lower levels of TC and TG were associated with increased plasma Sr concentrations. The mean Sr concentrations in these groups ranged from 35.8 µg/L to 40.8 µg/L [50]. The exposure levels of Fe, Cr, and Sr in the above studies were higher than those obtained in the present study. The designated safe levels of Cd and Pb in adult blood were < 5 µg/L [51] and < 3.5 µg/dL [52], respectively. In this study, Cd concentrations in the participants’ plasma were lower than those in the aforementioned studies and within the safety criteria. Plasma levels of lead in the population in this study area are higher than the safe limit and may have adverse health effects, including damage to the hematopoietic and neurological systems. However, no adverse effects of lead on dyslipidemia were found in the present study.

In contrast to some recognized risk factors for dyslipidemia (e.g., obesity), studies on the effect of heavy metal elements on the occurrence of dyslipidemia still deserve further investigation [53]. The BKMR model used in this study is capable of assessing not only the interactions between individual metals but also the effect of whole-metal mixtures on dyslipidemia. BKMR results suggested the lack of an overall effect of metal mixtures on dyslipidemia, but an effect on dyslipidemia may be present in the female population. The effect of combined metal exposure on dyslipidemia was not observed in a cross-sectional study from the United States [54]. However, a study of dyslipidemia in adults found significant between-group differences in multiple heavy metal exposures in females [8]. There are very few studies on the overall effect of metal mixtures on dyslipidemia. Obviously, more research is needed to explore the specific mechanisms of the interaction between combined metal exposure and dyslipidemia.

## Study strengths and limitations

This study had the following advantages. First, the use of the BKMR analysis method enabled a more realistic estimation of the overall relationship between metal mixtures and dyslipidemia, probed the potential interactions between combined metal exposures, reflected the true combined effects between metal mixtures and dyslipidemia, and identified important mixture components. This was the first time that the association between metal mixtures and dyslipidemia was analyzed by using the BKMR model. Second, gender-stratified analysis allowed us to more accurately elucidate the relationship between metal mixtures and dyslipidemia under different exposure conditions.

Meanwhile, several limitations still existed in this study. First, as a cross-sectional study, its nature prevented us from determining the causal relationship between exposure to metal elements and dyslipidemia. Second, dyslipidemia is often associated with dietary habits [55], but this analysis did not include the dietary intake of different nutrients. Third, the arsenic in the plasma in this study was inorganic arsenic, but organic arsenic is more toxic to humans than inorganic arsenic. Therefore, other studies reporting the toxicity of organic arsenic are needed to complement the conclusions of this article.

## Conclusion

Combined metal exposure was negatively associated with dyslipidemia in females, with Zn making the greatest contribution. After controlling for the effects of other heavy metal factors, As was positively associated with dyslipidemia, and Zn may be negatively associated with dyslipidemia in females. Females with low plasma Zn levels are more likely to develop dyslipidemia and should receive more clinical attention in this population. Subsequent analyses will need to incorporate dietary factors in participants to further investigate the relationship between metal mixtures and dyslipidemia by controlling for population exposure to different metals.

## Data Availability

Please contact the authors for reasonable requests.
